# Pure 16q21q22.1 deletion in a complex rearrangement possibly caused by a chromothripsis event

**DOI:** 10.1186/1755-8166-6-29

**Published:** 2013-08-01

**Authors:** Rita Genesio, Valentina Ronga, Pia Castelluccio, Gennaro Fioretti, Angela Mormile, Graziella Leone, Anna Conti, Maria Luigia Cavaliere, Lucio Nitsch

**Affiliations:** 1 DMMBM, Universita' di Napoli Federico II, Naples, Italy; 2 UOSC Genetica Medica, AORN A. Cardarelli, Naples, Italy

**Keywords:** Complex insertional translocation, Chromothripsis, Array-CGH, Developmental and growth delay, Dysmorphic features, Partial monosomy 16q21q22.1, Congenital heart defects

## Abstract

**Background:**

Partial monosomies of chromosome 16q are rare and overlapping effects from complex chromosomal rearrangements often hamper genotype-phenotype correlations for such imbalances. Here, we report the clinical features of an isolated partial monosomy 16q21q22.1 in a boy with a complex *de novo* rearrangement possibly resulting from a chromothripsis event.

**Results:**

The patient presented with low birth weight, microcephaly, developmental delay, facial dysmorphisms, short stature, dysmorphic ears and cardiopathy. Standard and molecular cytogenetics showed a complex rearrangement characterised by a pericentromeric inversion in one of chromosomes 12 and an inverted insertional translocation of the 12q14q21.1 region, from the rearranged chromosome 12, into the q21q22.1 tract of a chromosome 16. Array-CGH analysis unravelled a partial 16q21q22.1 monosomy, localised in the rearranged chromosome 16.

**Conclusions:**

The comparison of the present case to other 16q21q22 monosomies contributed to narrow down the critical region for cardiac anomalies in the 16q22 deletion syndrome. However, more cases, well characterised both for phenotypic signs and genomic details, are needed to further restrict candidate regions for phenotypic signs in 16q deletions. The present case also provided evidence that a very complex rearrangement, possibly caused by a chromothripsis event, might be hidden behind a classical phenotype that is specific for a syndrome.

## Background

Interstitial deletions of chromosome 16 long arm are rare rearrangements with around 20 patients described so far [1-7]. In spite of some specific phenotypes associated with the extension and localisation of the deleted regions, a critical region for this deletion syndrome is not clearly defined, as both 16q12.1q13 and 16q22 deletions are associated with similar phenotypic signs [8]. Nevertheless, 16q22 deletion is a recognised OMIM [9] syndrome (#614541), associated with the deletion of the whole 16q22 band, presenting with failure to thrive in infancy, poor growth, delayed psychomotor development, hypotonia, and dysmorphic features, including large anterior fontanelle, high forehead, diastasis of the cranial sutures, broad nasal bridge, hypertelorism, low-set abnormal ears, and short neck. The phenotypic features and deletion sizes of the affected patients are variable, but deletion of 16q22 appears to be critical for manifestations of the syndrome [10]. Congenital heart defects (CHD), mainly septal defects and anomalies of the outflow tract, have been described in 50–60% of the cases and, with a lower frequency, kidney malformations and anterior anus have also been reported. A candidate region for CHD has been proposed in the 16q22 region [1].

Here, we present a case of 16q21q22.1 isolated deletion resulting from a complex chromosomal rearrangement. The proband showed microcephaly, dysmorphisms and CHD. We have compared clinical and cytogenetic findings of this case with those described in the literature to gain more accurate genotype-phenotype correlations.

### Clinical report

The patient is the second child of healthy parents. He was born preterm at 33.6 weeks of gestation with a weight of 2.17 kg, a head circumference of 29.5 cm and length of 45.5 cm (appropriate for gestational age).

**Figure 1:**
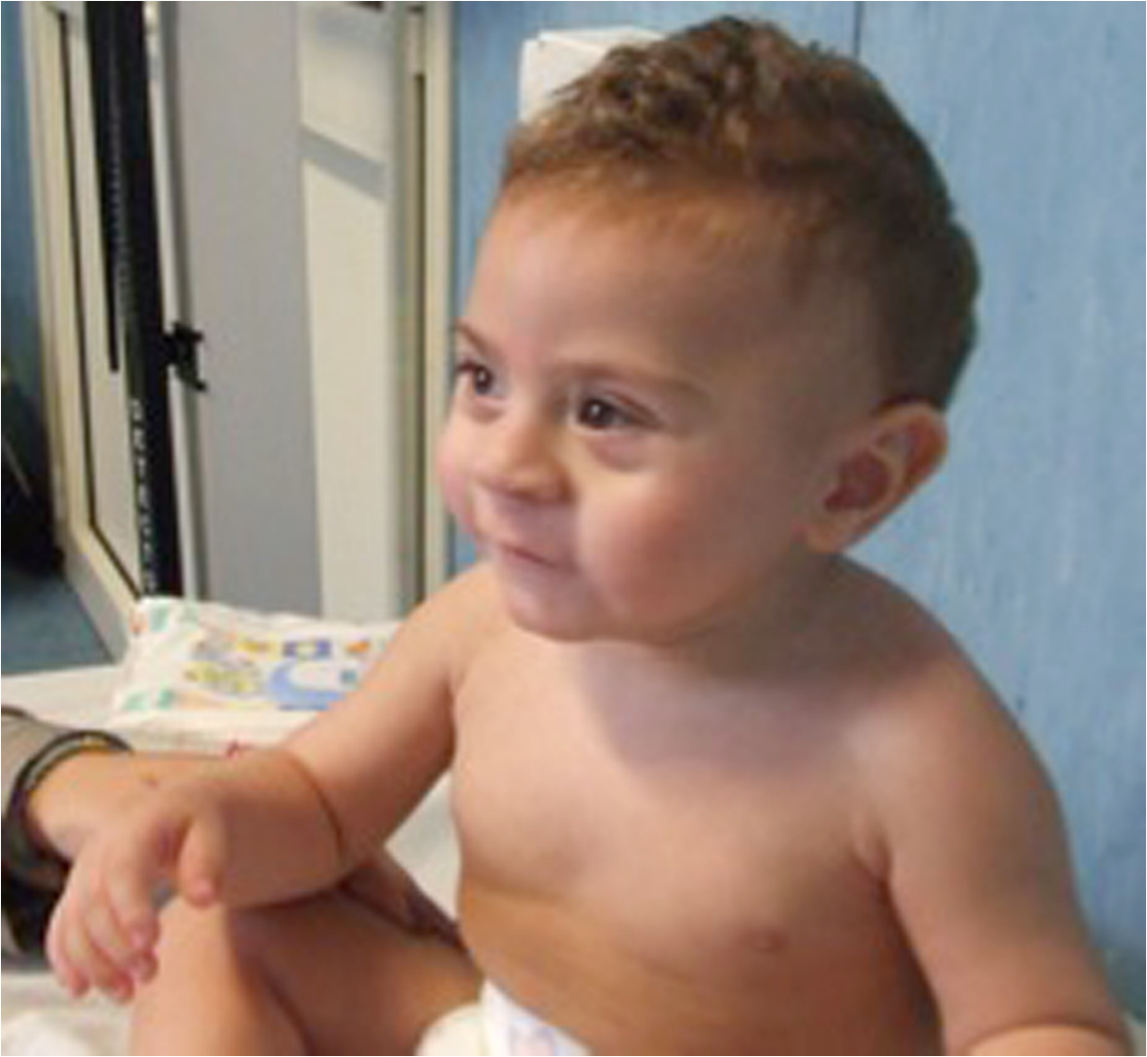
**The boy at the age of 1 year.** Note low set ears, epicanthus and thin upper-lip.

At the age of 2 months he suffered from an episode of respiratory distress and hyperkalaemia. An echocardiogram performed at that time revealed a CHD presenting with an atrial septal defect (ASD) and a patent *foramen ovale* (PFO). Notable findings on physical examination at 3 months and 8 days of age included: microcephaly (occipitofrontal circumference was 35.5 cm), sparse eyebrows, short ocular rhyme with proptosis, epicanthus, plans angiomas on middle forehead and on the eyelids, high philtrum, prominent and low set ears, thin upper-lip, simian crease, anterior anus, overlapping toes, and clinodactyly of the fifth fingers (Figure 1). Weight was 3.630 g (<3rd centile), length was 53 cm (<3rd centile).

## Results

**Figure 2:**
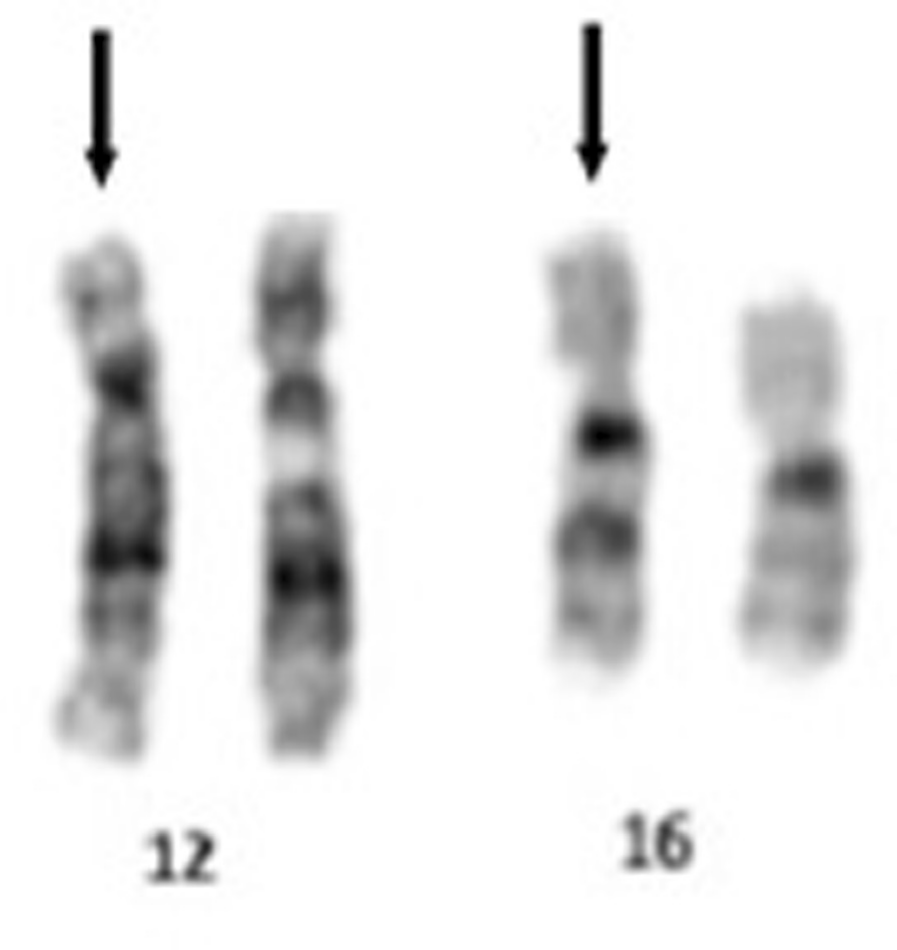
**G-Banding image of rearranged chromosomes 12 and 16.** G-Banded karyotype shows a pericentric inversion, from band p12 to q13, on one chromosome 12, an interstitial deletion of the region from 12q14 to 12q21 and additional material on one chromosome 16, within the bands q21q22. Arrows mark the derivative chromosomes.

Cytogenetic analysis of the proband, performed at 550 bands on G-banded metaphases from peripheral blood lymphocytes, revealed a pericentric inversion of one chromosome 12, from band p12 to q13, an interstitial deletion of the region from q14 to q21 on the same chromosome and the presence of additional material on one of the homologues of chromosome 16, within the bands q21q22 (Figure 2). Parental karyotypes were normal.

**Figure 3:**
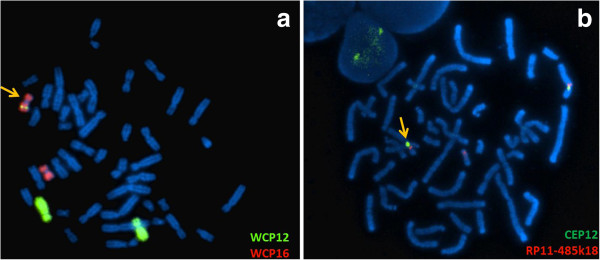
**Dual colour fluorescence *in situ *hybridization analysis of chromosome 16 and chromosome 12.** FISH analysis using wcp12 (green) and wcp16 (red) probes **(a)** or RP11-485K18 (red) BAC probe, mapping to 12p11.2 and CEP12 (green), used as control probe **(b)**, showed that the additional material present in the q arm of one chromosome 16 was derived from chromosome 12 and confirmed the pericentric inversion of one chromosome 12.

**Figure 4:**
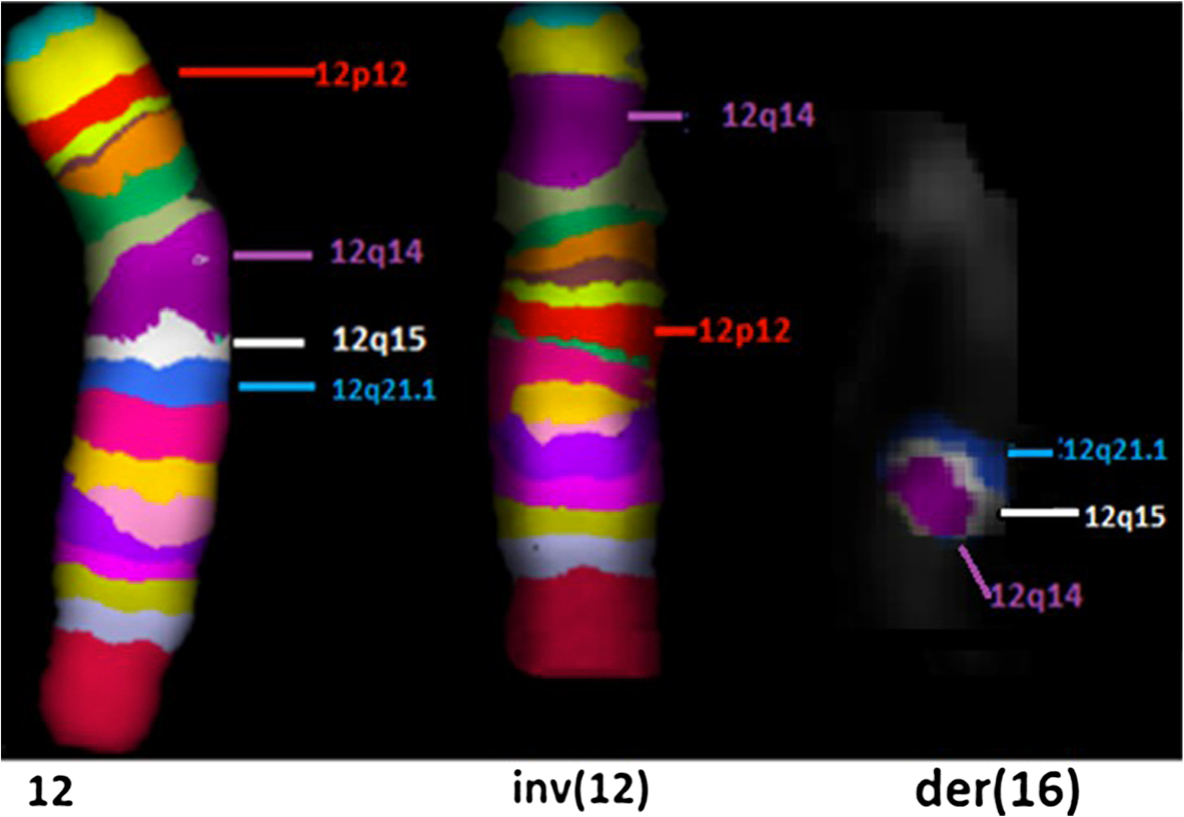
**High resolution multicolour banding (MCB).** High resolution MCB image of the normal chromosome 12 compared to the rearranged chromosome 12 shows that the pericentric inversion extended from the band 12p12 to the band 12q14. Chromosome 16 image shows an inverted insertion of the 12q14q21 region inside the tract 16q21q22.

Dual-colour-FISH using either the two Whole Chromosome Painting (WCP) probes for chromosomes 12 and 16 (Figure 3a) or the BAC probe RP11-485K18 (12p11.2) and CEP 12 (Figure 3b) revealed that the additional material present on the q arm of chromosome 16 was derived from chromosome 12 and confirmed the inversion. Multicolour banding (MCB) for chromosome 12 was performed to further characterise the rearrangement; this showed an inverted insertional translocation of the region 12q14q21 into the derivative chromosome 16, inside the tract 16q21q22. MCB confirmed that the pericentric inversion extended from the band 12p12 to the band 12q14 (Figure 4).

**Figure 5:**
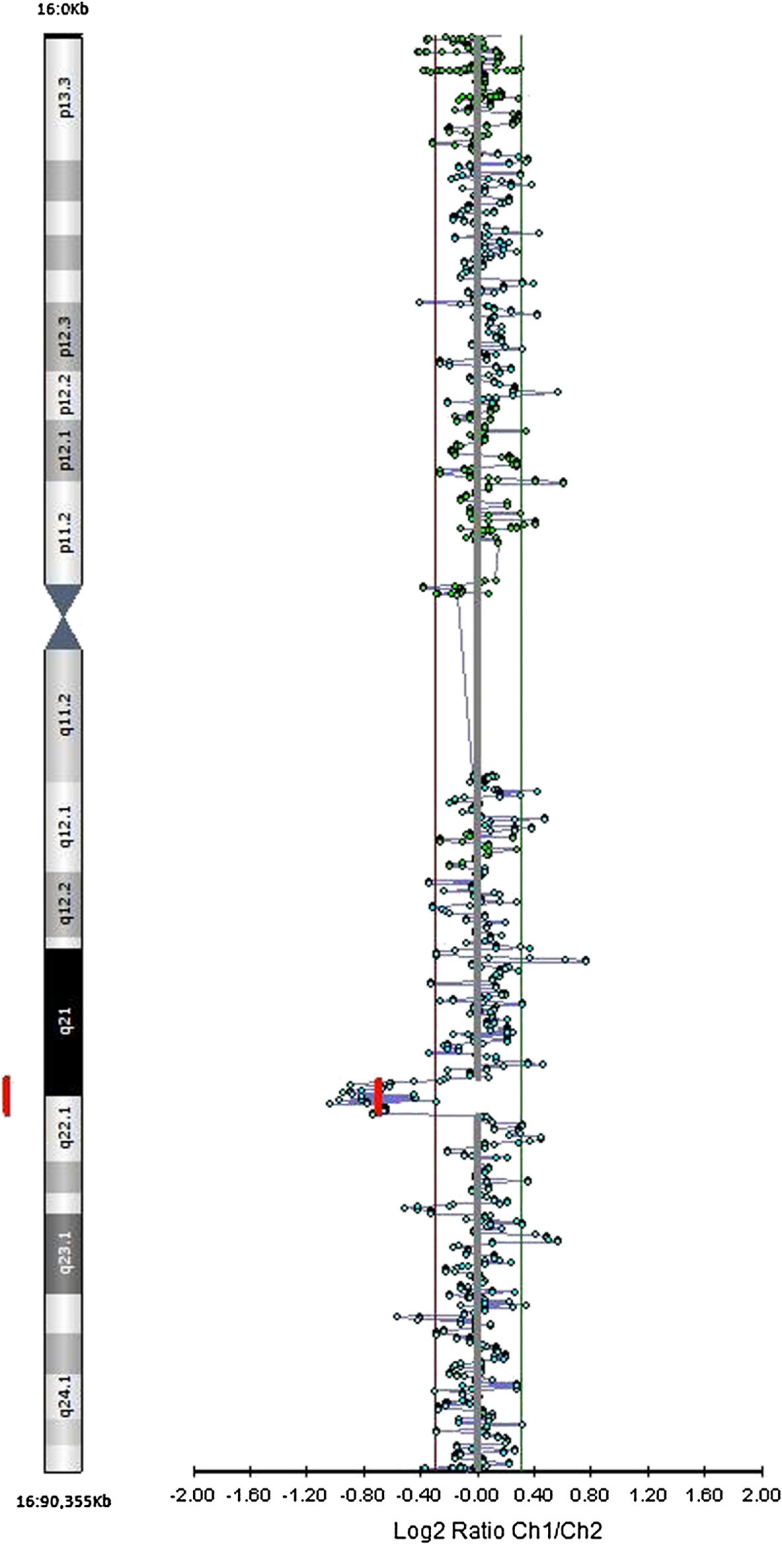
**Array-CGH analysis.** The analysis shows a partial deletion of the 16q21q22.1 tract, spanning approximately 2.1 Mb (chr16:65713516–67842736 [Hg19]), involving the region in which the 12q14q21.1 tract was inserted.

Finally, Oligo-array CGH analysis identified a 16q21q22.1 deletion, spanning approximately 2.1 Mb (chr16:65,713,516-67,842,736 [Hg19]), involving the region in which the 12q14q21.1 tract was inserted (Figure 5).

The complete chromosomal characterisation according to ISCN 2013 [11] was as follows:

46,XY,inv(12)(pter- > p13::q14- > p12::q21- > qter),ins(16;12)(16pter- > 16q21::12q21.1- > 12q14::16q22.1- > 16qter).arr [hg19]16q21q22.1(65,713,516-67,842,736)×1.

## Discussion

Few cases of 16q21q22 have been reported so far. The study of genotype-phenotype correlations in these cases was hampered either by the overlapping effects from associated chromosomal rearrangements or by the poor molecular characterisation of the rearrangements (only 2 cases out of 21 were characterised by array-CGH). The patient described herein presented an isolated partial monosomy 16q21q22.1 without additional chromosomal imbalances, as assessed by array-CGH. He shows the typical symptoms of 16q22 deletion syndrome, such as low birth weight, global developmental delay, short stature, microcephaly, low set dysmorphic ears, mild facial dysmorphisms and CHD. The accurate molecular characterisation performed here might contribute to better define candidate regions for specific phenotypic signs, such as CHD. Yamamoto et al. [1] compared their proband presenting CHD to a group of 13 previously described patients, with overlapping deletion encompassing the 16q21q22 tract. Nine patients out of 14 (64%) showed CHD, mainly related to endocardial cushion and outflow-tract defects. By analysing the overlapping regions, the authors hypothesised that a candidate region for CHD could be identified between the middle part of 16q21 and the middle of 16q22 (from 62,000,000 to 68,000,000) [1]. The 16q21 band, with a very low gene density, is not the major candidate, as it represents a wide region of euchromatic copy number variations (CNV) without phenotypic effect [12]. This hypothesis was reinforced by Coussement et al. [3] who reported a 5.8 Mb deletion at 16q21 (from 59,934,099 to 65,702,372–Hg19) without abnormal phenotype in three generations of a family.

**Figure 6:**
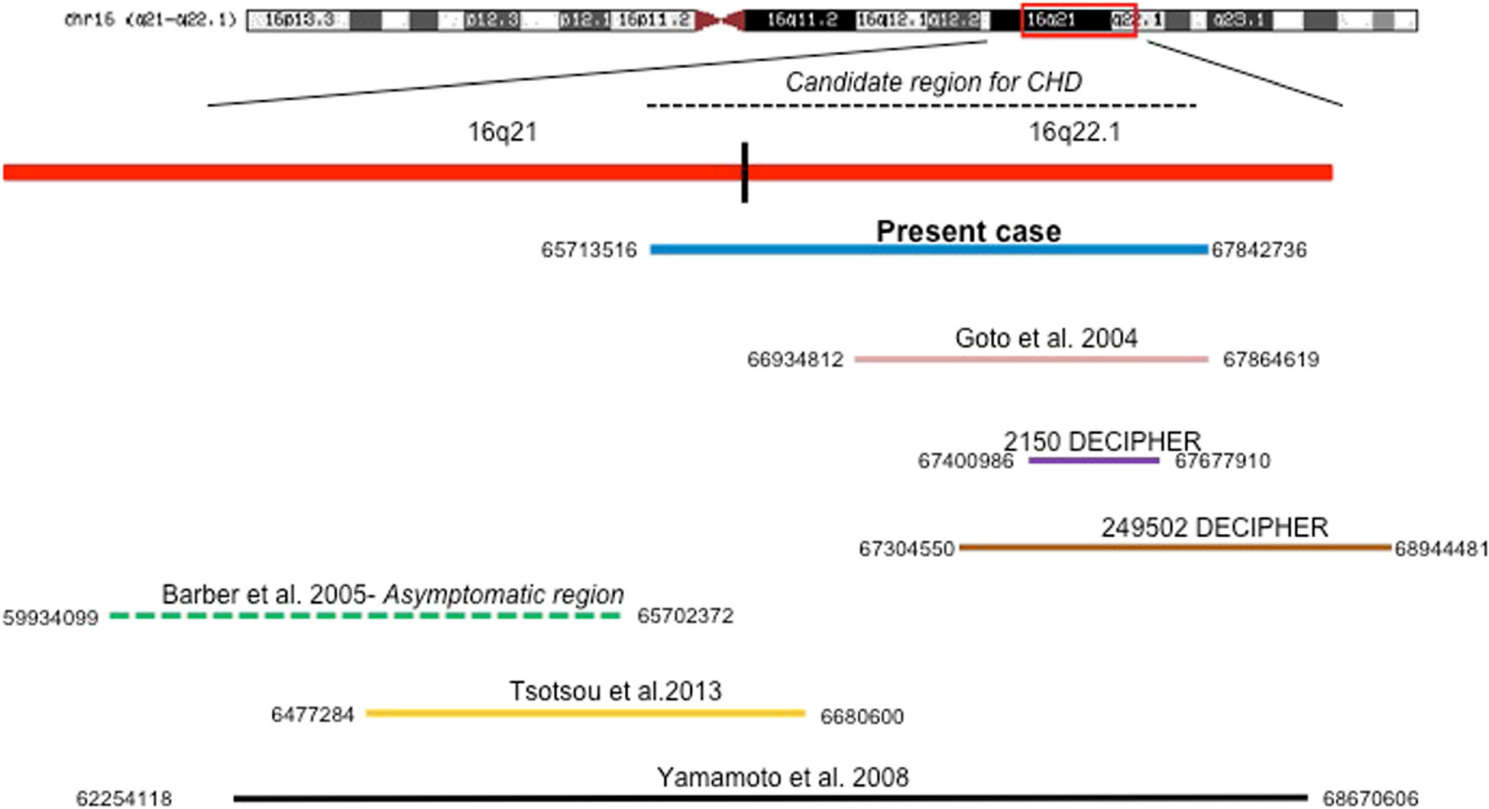
**Overlapping of 16q21q22 deletions.** The figure shows the present case (blue line), the patients described by Yamamoto et al. [1] (black line), by Goto et al. [13] (pink line), by Tsoutsou et al. [5] (yellow line), the DECIPHER cases 2150 (purple line) and 249502 (brown line) and the asymptomatic region described by Coussement et al. [3] (green line).

We here compare the deletion tract of the present case to the 3 patients described [1,5,13], to patients 2150 and 249502 from the DECIPHER database [14] and to asymptomatic subjects described by Coussement [3] (Figure 6), whose deletions are partially overlapping. Clinical signs of the patients carrying phenotypic anomalies are compared in Table 1.

**Table 1:** Clinical phenotype of syndromic patients with 16q deletion syndrome

		**Yamamoto et al.**[1]	**Tsoutsou et al.**[5]	**Goto et al.**[13]	**DECIPHER 2150**	**DECIPHER 249502**	**Present case**
**Growth**	Small for dates	**+**					**+**
	Postnatal <3rd centile	**+**	**+**	**+**			**+**
	Microcephaly	**+**	**+**	**+**			**+**
**Central nervous system and development**	Psychomotor retardation	**+**	**+**	**mild**	**+**	**+**	**+**
**Head**	Large anterior fontanelle	**+**	**+**	**+**			
	High forehead	**+**	**+**				
	Diastasis cranial sutures	**-**		**+**			
**Dysmorphisms**	Broad flat nasal bridge	**+**	**+**			**+**	
	Hypertelorism	**+**					
	Epicanthal fold		**+**	**+**			**+**
	Low set dysmorphic ears	**+**	**+**		**+**		**+**
	Anomalies of palpebral fissures	**+**				**+**	**+**
	Upward slanting palpebral fissures	**+**		**+**			**+**
	Micrognathia	**+**	**+**				**+**
**Thorax and abdomen**	Congenital heart defects	**TOF**					**ASD + PFO**
	Ectopic anus	**-**					**+**
**Extremities**	Flexed fingers	**-**	**+**				
	Bilateral simian creases	**+**	**+**				**+**
	Malposition of toes	**+**				**+**	**+**

*TOF* Tetralogy of Fallot, *ASD* Atrial Septal Defect, *PFO* Patent *Foramen Ovale*.

Patient 2150 from DECIPHER, with psychomotor retardation and dysmorphic features, showed the smallest 16q22.1 deletion so far described (67,400,986-67,677,910–Hg19), sharing with other cases a region spanning 200Kb, which includes only 8 genes. One of these genes, *HSD11B2*, encodes the type II isoform of 11-beta-hydroxycorticosteroid dehydrogenase, a microsomal enzyme complex that is responsible for inactivation of biologically active cortisol. A moderate deficiency of the gene was hypothesised to predispose to low birth weight [15], which is a very common feature of patients with interstitial 16q deletions, including the present case. The presence of dysmorphic ears in the case 2150 also suggests that the haploinsufficiency of transcripts mapping to the 200Kb deleted tract might be responsible for the ear dysmorphic features that have been described in almost all of the 16q22 deleted patients.

As the case described here presented CHD, we have re-evaluated a possible critical region for CHD comparing the 16q deleted tract of the proband to that of patients described by Yamamoto et al. [1] and to that described for the asymptomatic subjects [3]. This analysis suggested that the critical region responsible for CHD might span about 2 Mb, from the distal region of 16q21 to the 16q22.1 sub-band (chr16:65,700,000-67,845,000). No genes involved in cardiogenesis have been identified in this region.

Many molecular mechanisms have been proposed to explain the origin of the complex chromosomal rearrangements (CCRs) that are generated by a number of recombination events, resulting either from errors in the repair processes or from a defect of the recombination system. In addition to the mechanisms proposed such as non-allelic homologous recombination (NAHR), non-homologous end joining (NHEJ), fork stalling and template switching (FoSTeS) [16] and microhomology mediated break-induced replication (MMBIR) [17], recently, a new phenomenon, called chromothripsis, has been described as a cause of CCR, through clustered double-stranded DNA breaks and non-homologous repair mechanisms [18]. This phenomenon causes the localised crushing of one or more chromosomes and subsequent incorrect reassembly of the parts arising through NHEJ or MMBIR mechanisms, with sporadic deletions (including small deletions of only a few hundred base pairs) [19].

## Conclusions

**Figure 7:**
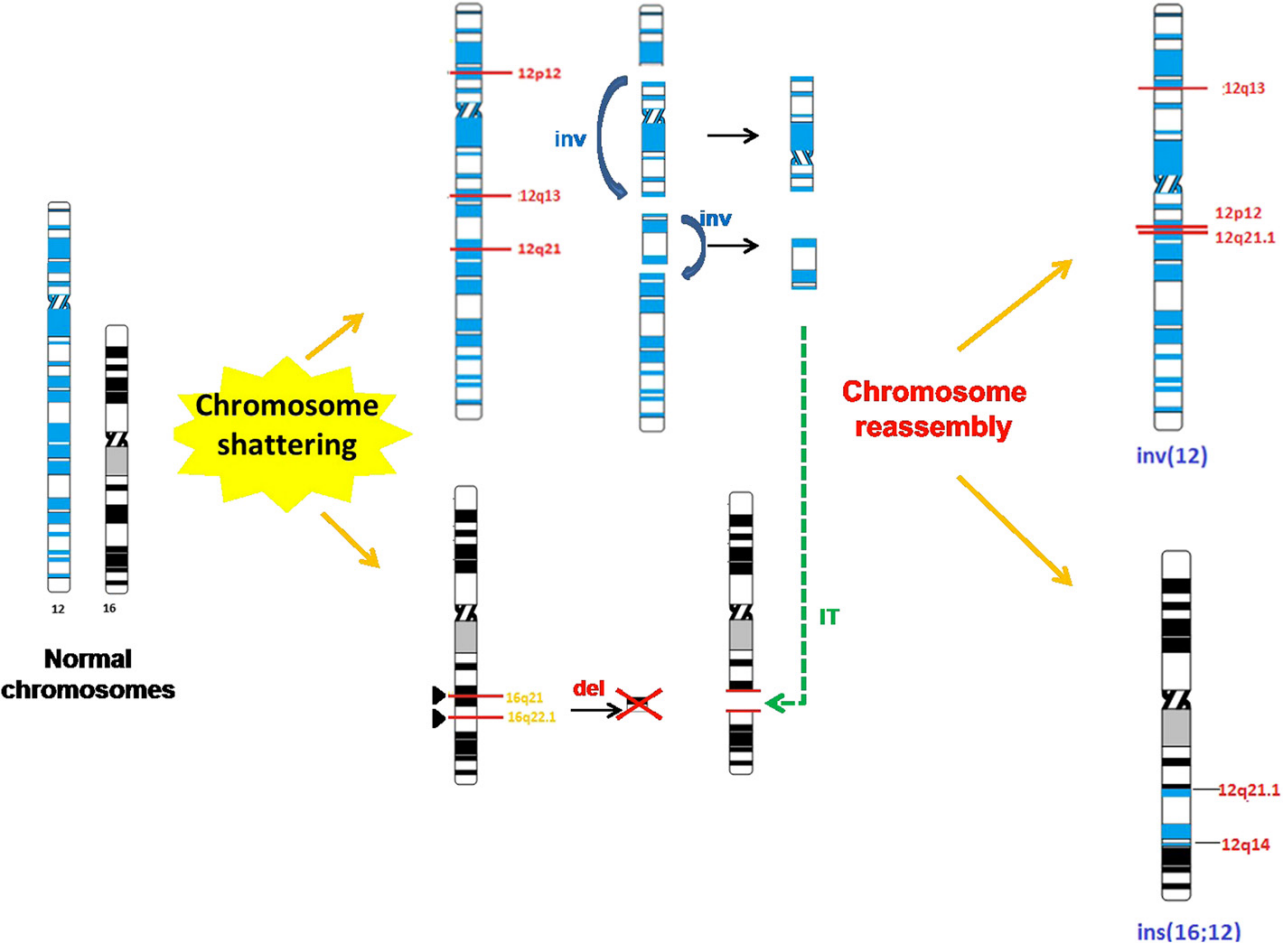
**Schematic representation of possible mechanisms cause of the complex chromosomal rearrangement in the proband.** DNA double-strand breaks are formed as a result of "chromosome shattering" (triangles blacks) with the corresponding formation of chromosomal fragments that are rearranged through the phenomenon of "chromosome reassembly". The result is the formation of inversions (inv, blue arrows), deletions (the black arrow) and insertional translocation (IT, green dashed arrow) that originate the two derivative chromosomes: chromosome 12 with a pericentric inversion and deletion of 12q14q21.1 and chromosome 16 with the concomitant insertion of the 12q14q21.1 region with inverted sense in place of the 16q21-22.1 region that is deleted.

The case that we have characterised, with 5 breakpoints, one inversion, 2 deletions and the inverted insertion of one of the deleted tracts within a non-homologous chromosome, meets the criteria to be ascribed to an event of constitutional chromothripsis, as hypothesised in Figure 7. The confirmation of such hypothesis will come from further studies by breakpoint sequencing.

Notably, in spite of the complex genomic rearrangement resulting from the catastrophic events, the present case showed only typical signs of a single syndrome, demonstrating that even in the case of classical phenotypes we cannot exclude very complex rearrangements. This highlights that performing either array-CGH or karyotyping alone might give incomplete information, making it advisable, when possible, to perform both investigations.

The comparison of the present case to other 16q21q22 monosomies narrowed down the candidate critical region for cardiac anomalies in the 16q22 deletion syndrome.

## Methods

### Cytogenetics and FISH

Peripheral blood from the patient was cultured for 72 hr in the presence of phytohaemagglutinin. Metaphase spread preparations and GTG-banding were performed according to standard methods. A total of 20 metaphase cells were analysed. Karyotypes were described according to the International System for Human Cytogenetic Nomenclature.

FISH analysis was performed using Whole Chromosome Painting (WCP) for chromosomes 12 and 16. The Bacterial Artificial Chromosome (BAC) probe RP11-485 K18, specific for band 12p11.2, was selected from the UCSC genome browser [20] (UC Santa Cruz, USA, assembly March 2006) and provided by Professor M. Rocchi (rocchi@biologia.uniba.it). FISH experiments were performed on metaphase spreads according to standard protocols.

MCB was performed using the multicolour banding DNA Probe Kit based on microdissection derived region-specific libraries for chromosome 12 (MetaSystems, Arese MI).

Fluorescent images were analysed using a fluorescence microscope (AxioImager.Z1 mot, Zeiss) with ISIS software imaging system (MetaSystems, Altlussheim, Germany) for image capturing and processing.

### Array-CGH

A 4×44 CytoChip™ array with ISCA design (BlueGnome Ltd; Cambridge, UK) was used in accordance with manufacturer’s guidelines for the whole genome screening. The arrays were scanned using the InnoScan 710 and analysed using BlueFuse for Microarrays 3.5 software (BlueGnome, Cambridge, UK), referring to Hg19 Genome Assembly (NCBI Build GRCh37). Copy number variations were classified according to the Database of Genomic Variants [21], the DECIPHER Database [14] and the UCSC Genome Browser [20].

## Consent

Written informed consent was obtained from the patient’s parents for publication of this case report and any accompanying images.

## Abbreviations

CGH: Comparative Genomic Hybridisation; ISCA: International Standards for Cytogenomic Arrays; NCBI: National Center for Biotechnology Information; OMIM: Online Mendelian Inheritance in Man; BAC: Bacterial Artificial Chromosome; CEP: Chromosome Enumeration Probes; UCSC: University of California Santa Cruz; DECIPHER: Database of Chromosomal Imbalance and Phenotype in Humans using Ensembl Resources.

## Competing interests

The authors declare that they have no competing interests.

## Authors’ contributions

RG and VR equally contributed to the manuscript, carrying out the molecular genetic studies and drafting the manuscript. PC and MLC analysed the proband's phenotype, describing clinical features. GF, AM and GL participated to the cytogenetic and molecular cytogenetic characterisation and performed a literature survey. AC helped to draft the manuscript and curated its submission. LN has been involved in revising the manuscript critically for important intellectual content and has given final approval of the version to be published. All authors read and approved the final manuscript.

## References

[B1] Yamamoto T Dowa Y Ueda H Kawataki M Asou T Sasaki Y Harada N Matsumoto N Matsuoka R Kurosawa K Tetralogy of Fallot associated with pulmonary atresia and major aortopulmonary collateral arteries in a patient with interstitial deletion of 16q21-q22.1 Am J Med Genet A 2008 146A 1575 1580 10.1002/ajmg.a.32204 18470894

[B2] Borgatti R Marelli S Bernardini L Novelli A Cavallini A Tonelli A Bassi MT Dallapiccola B Bilateral frontoparietal polymicrogyria (BFPP) syndrome secondary to a 16q12.1-q21 chromosome deletion involving GPR56 gene Clin Genet 2009 76 573 576 10.1111/j.1399-0004.2009.01262.x 19807741

[B3] Coussement A Lochu P Dupont JM Choiset A Inherited interstitial 16q21 deletion of 5.8 Mb without apparent phenotypic effect in three generations of a family: an array-CGH study Am J Med Genet A 2011 155A 2597 2600 21910236 10.1002/ajmg.a.34210

[B4] Palka Bayard de Volo C Alfonsi M Gatta V Novelli A Bernardini L Fantasia D Antonucci I Angelucci D Zori R Stuppia L Chiarelli F Calabrese G 16q22.1 microdeletion detected by array-CGH in a family with mental retardation and lobular breast cancer Gene 2012 498 328 331 10.1016/j.gene.2012.01.028 22326525

[B5] Tsoutsou E Tzetis M Giannikou K Syrmou A Oikonomakis V Kosma K Kanioura A Kanavakis E Fryssira H Array-CGH revealed one of the smallest 16q21q22.1 microdeletions in a female patient with psychomotor retardation Eur J Paediatr Neurol 2013 17 316 320 10.1016/j.ejpn.2012.12.004 23352671

[B6] Pagnamenta AT Khan H Walker S Gerrelli D Wing K Bonaglia MC Giorda R Berney T Mani E Molteni M Pinto D Le Couteur A Hallmayer J Sutcliffe JS Szatmari P Paterson AD Scherer SW Vieland VJ Monaco AP Rare familial 16q21 microdeletions under a linkage peak implicate cadherin 8 (CDH8) in susceptibility to autism and learning disability J Med Genet 2011 48 48 54 10.1136/jmg.2010.079426 20972252 PMC3003876

[B7] Knoblauch H Thiel G Tinschert S Körner H Tennstedt C Chaoui R Kohlhase J Dixkens C Blanck C Clinical and molecular cytogenetic studies of a large de novo interstitial deletion 16q11.2-16q21 including the putative transcription factor gene SALL1 J Med Genet 2000 37 389 392 10.1136/jmg.37.5.389 10905896 PMC1734584

[B8] Callen DF Eyre H Lane S Shen Y Hansmann I Spinner N Zackai E McDonald-McGinn D Schuffenhauer S Wauters J Van Thienen MN Van Roy B Sutherland GR Haan EA High resolution mapping of interstitial long arm deletions of chromosome 16: relationship to phenotype J Med Genet 1993 30 828 832 10.1136/jmg.30.10.828 8230159 PMC1016564

[B9] Online Mendelian Inheritance in Man [http://www.ncbi.nlm.nih.gov/omim]

[B10] Fujiwara M Yoshimoto T Morita Y Kamada M Interstitial deletion of chromosome 16q: 16q22 is critical for 16q-syndrome Am J Med Genet 1992 43 561 564 10.1002/ajmg.1320430311 1605249

[B11] Shaffer LG McGowan-Jordan J Schmid M ISCN (2013): An International System for Human Cytogenetic Nomenclature 2013 Basel: S. Karger

[B12] Barber JC Directly transmitted unbalanced chromosome abnormalities and euchromatic variants J Med Genet 2005 42 609 629 10.1136/jmg.2004.026955 16061560 PMC1736115

[B13] Goto T Aramaki M Yoshihashi H Nishimura G Hasegawa Y Takahashi T Ishii T Fukushima Y Kosaki K Large fontanelles are a shared feature of haploinsufficiency of RUNX2 and its co-activator CBFB Congenit Anom (Kyoto) 2004 44 225 229 10.1111/j.1741-4520.2004.00043.x 15566413

[B14] Database of Chromosomal Imbalance and Phenotype in Humans using Ensembl Resources [http://decipher.sanger.ac.uk] 10.1016/j.ajhg.2009.03.010 PMC2667985 19344873

[B15] Mune T Rogerson FM Nikkila H Agarwal AK White PC Human hypertension caused by mutations in the kidney isozyme of 11 beta-hydroxysteroid dehydrogenase Nat Genet 1995 10 394 399 10.1038/ng0895-394 7670488

[B16] Gu W Zhang F Lupski JR Mechanisms for human genomic rearrangements Pathogenetics 2008 1 4 10.1186/1755-8417-1-4 19014668 PMC2583991

[B17] Hastings PJ Ira G Lupski JR A microhomology-mediated break-induced replication model for the origin of human copy number variation PLoS Genet 2009 5 e1000327 10.1371/journal.pgen.1000327 19180184 PMC2621351

[B18] Liu P Erez A Nagamani SC Dhar SU Kolodziejska KE Dharmadhikari AV Cooper ML Wiszniewska J Zhang F Withers MA Bacino CA Campos-Acevedo LD Delgado MR Freedenberg D Garnica A Grebe TA Hernandez-Almaguer D Immken L Lalani SR McLean SD Northrup H Scaglia F Strathearn L Trapane P Kang SH Patel A Cheung SW Hastings PJ Stankiewicz P Lupski JR Bi W Chromosome catastrophes involve replication mechanisms generating complex genomic rearrangements Cell 2011 146 889 903 10.1016/j.cell.2011.07.042 21925314 PMC3242451

[B19] Kloosterman WP Tavakoli-Yaraki M van Roosmalen MJ van Binsbergen E Renkens I Duran K Ballarati L Vergult S Giardino D Hansson K Ruivenkamp CA Jager M van Haeringen A Ippel EF Haaf T Passarge E Hochstenbach R Menten B Larizza L Guryev V Poot M Cuppen E Constitutional chromothripsis rearrangements involve clustered double-stranded DNA breaks and nonhomologous repair mechanisms Cell Rep 2012 1 648 655 10.1016/j.celrep.2012.05.009 22813740

[B20] University of California Santa Cruz Genome Browser [http://genome.ucsc.edu]

[B21] Database of Genomic Variants [http://projects.tcag.ca/variation/]

